# Validation of the Martin Method for Estimating Low-Density Lipoprotein Cholesterol Levels in Korean Adults: Findings from the Korea National Health and Nutrition Examination Survey, 2009-2011

**DOI:** 10.1371/journal.pone.0148147

**Published:** 2016-01-29

**Authors:** Jongseok Lee, Sungok Jang, Heejeong Son

**Affiliations:** 1 School of Business Administration, Hallym University, Chuncheon, Korea; 2 Korea Association of Health Promotion Gwangju-Jeonnam Branch, Gwangju, Korea; 3 Department of Anesthesiology, Kangwon National University School of Medicine, Chuncheon, Korea; University of Insubria, ITALY

## Abstract

Despite the importance of accurate assessment for low-density lipoprotein cholesterol (LDL-C), the Friedewald formula has primarily been used as a cost-effective method to estimate LDL-C when triglycerides are less than 400 mg/dL. In a recent study, an alternative to the formula was proposed to improve estimation of LDL-C. We evaluated the performance of the novel method versus the Friedewald formula using a sample of 5,642 Korean adults with LDL-C measured by an enzymatic homogeneous assay (LDL-C_D_). Friedewald LDL-C (LDL-C_F_) was estimated using a fixed factor of 5 for the ratio of triglycerides to very-low-density lipoprotein cholesterol (TG:VLDL-C ratio). However, the novel LDL-C (LDL-C_N_) estimates were calculated using the N-strata-specific median TG:VLDL-C ratios, LDL-C_5_ and LDL-C_25_ from respective ratios derived from our data set, and LDL-C_180_ from the 180-cell table reported by the original study. Compared with LDL-C_F_, each LDL-C_N_ estimate exhibited a significantly higher overall concordance in the NCEP-ATP III guideline classification with LDL-C_D_ (*p*< 0.001 for each comparison). Overall concordance was 78.2% for LDL-C_F_, 81.6% for LDL-C_5_, 82.3% for LDL-C_25_, and 82.0% for LDL-C_180_. Compared to LDL-C_5_, LDL-C_25_ significantly but slightly improved overall concordance (*p* = 0.008). LDL-C_25_ and LDL-C_180_ provided almost the same overall concordance; however, LDL-C_180_ achieved superior improvement in classifying LDL-C < 70 mg/dL compared to the other estimates. In subjects with triglycerides of 200 to 399 mg/dL, each LDL-C_N_ estimate showed a significantly higher concordance than that of LDL-C_F_ (*p*< 0.001 for each comparison). The novel method offers a significant improvement in LDL-C estimation when compared with the Friedewald formula. However, it requires further modification and validation considering the racial differences as well as the specific character of the applied measuring method.

## Introduction

Because low-density lipoprotein cholesterol (LDL-C) is a major modifiable risk factor for cardiovascular disease (CVD) [1], its accurate assessment is important for therapeutic decisions. In routine clinical practice worldwide, it is typically calculated using the Friedewald formula [2]. In the Korea National Health Screening Program (KNHSP), LDL-C is calculated but not directly measured when triglyceride levels are lower than 400 mg/dL. Of 11,380,246 participants who examined their triglyceride levels in the 2013 KNHSP, there were 11,143,810 persons (98%) with triglyceride levels under 400 mg/dL [3], implying that LDL-C was directly measured for only 2% of the participants. From the outset, the formula’s inaccuracies at triglyceride levels ≥400 mg/dL were recognized by Friedewald et al. [4]. However, even when triglyceride levels are under 400 mg/dL, a number of studies have suggested that LDL-C estimates by the formula (LDL-C_F_) underestimate LDL-C and thus misclassify CVC risk [5–8], particularly in individuals with high levels of triglycerides [5–7] and LDL-C less than 70 mg/dL [8].

In a recent study by Martin et al. [9], an alternative to the Friedewald formula was proposed to improve estimation of LDL-C at triglyceride levels under 400 mg/dL. The Friedewald equation calculates LDL-C as LDL-C_F_ = [total cholesterol]–[high-density lipoprotein cholesterol (HDL-C)]–[triglycerides / 5], where the final term is the estimate of very-low-density lipoprotein cholesterol (VLDL-C). This equation therefore uses a fixed factor of 5 for the ratio of triglycerides to VLDL-C (TG:VLDL-C); however, the Martin equation applies an adjustable factor determined as the N-strata-specific median TG:VLDL-C ratio based on triglyceride and non-HDL-C concentrations to estimate the novel LDL-C (LDL-C_N_). Compared with LDL-C_F_, LDL-C_N_ was reported to be closer to directly measured LDL-C (LDL-C_D_) and improved concordance in guideline risk classification with LDL-C_D_, especially at LDL-C less than 70 mg/dL.

For adoption of the novel method, external validation is required in independent populations based on various races and the use of other laboratory techniques. Martin et al. [9] derived the strata-specific TG:VLDL-C ratios using a large cohort of United States patients with LDL-C measured by the vertical auto profile (VAP) method. To our knowledge, none have validated the Martin equation in a Korean population. We therefore evaluated the performance of the LDL-C_N_ estimation method using an independent sample, nationally representative data from the Korea National Health and Nutrition Examination Survey (KNHANES) conducted from 2009 through 2011, in which LDL-C was directly measured by an enzymatic homogenous assay.

## Materials and Methods

This study was reviewed and approved by the Kangwon National University Hospital’s institutional review board (KNUH-2015-07-012). The Korea center of Disease Control and Prevention received the informed consent from all participants. The data are publicly available in website [https://knhanes.cdc.go.kr/knhanes/index.do].

### Study population

This study was performed using data from the 2009–2011 KNHANES[10, 11]. The KNHANES is a nationwide, population-based, cross-sectional survey conducted by the Korean Center for Disease Control and Prevention since 1998. This survey used a stratified, multistage, clustered probability sampling method to select a representative sample of the non-institutional, civilian Korean population. All participants in this survey provided written informed consent.

The number of KNHANES participants was 10,533 in 2009; 8,958 in 2010; and 8,518 in 2011. Among those who participated in the survey, this study included 5,790 adults aged 20 years and older who completed examination of total cholesterol, HDL-C, triglycerides, and LDL-C_D_. Additionally, 198 subjects with triglyceride levels of 400 mg/dL and higher were excluded, leaving 5,642 subjects (2,723 males and 2,919 females) for the main analysis. The number of subjects by year was as follows: 1,881 in 2009; 1,867 in 2010; and 1,894 in 2011.

### Measurements and Estimations

Blood samples were drawn from the antecubital vein of each subject the morning after fasting for at least 8 hours. Serum lipid concentrations were directly measured by an enzymatic method using an automated analyzer (Hitachi Automatic Analyzer 7600, Hitachi, Tokyo, Japan), including measurements of total cholesterol (Pureauto S CHO-N; Sekisui Medical, Tokyo, Japan), HDL-C (Cholestest N HDL; Sekisui Medical), and triglycerides (Pureauto S TG-N; Sekisui Medical). Serum LDL-C concentrations (LDL-C_D_) were directly measured using an enzymatic homogenous assay with Cholestest-LDL (Sekisui Medical). Non-HDL-C was calculated by subtracting HDL-C from total cholesterol. VLDL-C was calculated using the following subtraction equation: VLDL-C = [non-HDL-C]–[LDL-C_D_].

LDL-C_F_ was estimated as [non-HDL-C]–[triglycerides / 5] [4]. LDL-C_180_ was calculated as [non-HDL-C]–[triglycerides / AF], where AF is an adjustable factor in the 180-cell table described by Martin et al. [9]. In addition, two LDL-C_N_ estimates were calculated using strata-specific TG:VLDL-C ratios derived from our data set: LDL-C_5_ based on 5 strata of triglycerides and LDL-C_25_ based on 25 strata of triglyceride and non-HDL-C levels.

### Statistical analysis

The data were analyzed using SPSS for Windows (version 21.0; SPSS Inc., Chicago, IL, USA). All statistical outcomes were based on two-sided tests, and a *p-*value less than 0.05 was considered statistically significant. Data are summarized as median and range for continuous variables, and frequency and percentage for categorical variables. To compare the two triglyceride groups (subjects with triglycerides ≤ 400 mg/dL and those with > 400 mg/dL), the median test was used for continuous variables, and Fisher’s exact test was used for categorical variables. Multiple regression analysis was performed to investigate the extent to which VLDL-C was explained by triglycerides, non-HDL-C, HDL-C, and age.

When triglyceride levels were lower than 400 mg/dL, the Wilcoxon signed rank test was conducted to examine the difference between each estimated LDL-C and LDL-C_D_ value, and to determine whether LDL-C_N_ estimates more closely approximated LDL-C_D_ compared with LDL-C_F_. The median test was used to compare the median TG:VLDL-C ratios among five strata based on triglyceride levels. One-way analysis of variance (ANOVA) was performed for multiple comparison of the mean VLDL-C: TG ratios among the triglyceride strata.

Directly measured and estimated LDL-C values were classified according to the National Cholesterol Education Program Adult Treatment Panel III (NCEP-ATP III) guideline cutoffs[1] of 70, 100, 130, 160, and 190 mg/dL. Concordance in classification between LDL-C estimates and LDL-C_D_ was examined through cross-tabulations by LDL-C categories in subjects with triglycerides lower than 400 mg/dL. McNemar’s exact test for correlated proportions was performed to compare overall concordance between LDL-C estimates.

## Results

Table 1 shows sex, age, and lipid characteristics of the sample population overall and by triglyceride group. Of 5,790 samples, only 148 subjects (2.6%) had triglyceride levels of 400 mg/dL and higher. All variables except for median age were significantly different between triglyceride groups. 5,642 subjects with triglyceride levels under 400 mg/dL were mostly middle-aged [median, 45 years; interquartile range (IQR), 33–57 years] and evenly distributed by sex [48.3% male]. In this triglyceride group, the median TG:VLDL-C ratio was 4.9, the IQR was 3.7–6.1, the 5th to 95th percentile was 2.3 to 8.4, the 1st to 99th percentile was 1.6 to 11.5, and the full range was 0.6 to 285.7.

**Table 1:** Characteristics of the study population by triglyceride strata.

	Number (Percentage) or Median (IQR) *			
	Total	TG < 400 mg/dL	TG ≥ 400 mg/dL	
Variable	(*n* = 5,790)	(*n* = 5,642)	(*n* = 148)	*p*-value^†^
**Age, years**	45 (33–57)	45 (33–57)	48 (39–57)	0.052
**Sex, *n* (%)**				< 0.001
Male	2,841 (49.1)	2,723 (48.3)	118 (79.7)	
Female	2,949 (50.9)	2,919 (51.7)	30 (20.3)	
**Cholesterol, mg/dL**				
Total	184 (162–209)	184 (162–209)	217 (191–244)	< 0.001
HDL-C	47 (40–55)	48 (41–55)	37 (33–42)	< 0.001
Non-HDL-C	136 (113–162)	135 (113–161)	178 (154–203)	< 0.001
Direct LDL-C	110 (90–133)	111 (91–133)	96 (75–117)	< 0.001
VLDL-C	23 (17–31)	22 (17–30)	74 (63–99)	< 0.001
**Triglycerides, mg/dL**	117 (72–163)	106 (71–157)	505 (432–635)	< 0.001
**TG:VLDL-C ratio**	4.9 (3.8–6.2)	4.9 (3.7–6.1)	6.8 (6.1–7.9)	< 0.001
**VLDL-C:TG ratio**	0.20 (0.16–0.27)	0.21 (0.16–0.27)	0.15 (75–117)	< 0.001

IQR indicates interquartile range; TG, triglyceride; HDL-C, high-density lipoprotein cholesterol; Direct LDL-C, low-density lipoprotein cholesterol measured by the enzymatic homogeneous assay; VLDL-C, very low-density lipoprotein cholesterol; TG:VLDL-C ratio, ratio of TG to VLDL-C; VLDL-C:TG ratio, ratio of VLDL-C to TG.

* Data are expressed as medians (IQR) for continuous variables and frequencies (percentages) for categorical variables.

^†^ Statistical significance was assessed using Fisher’s exact test or median test.

**Fig 1:**
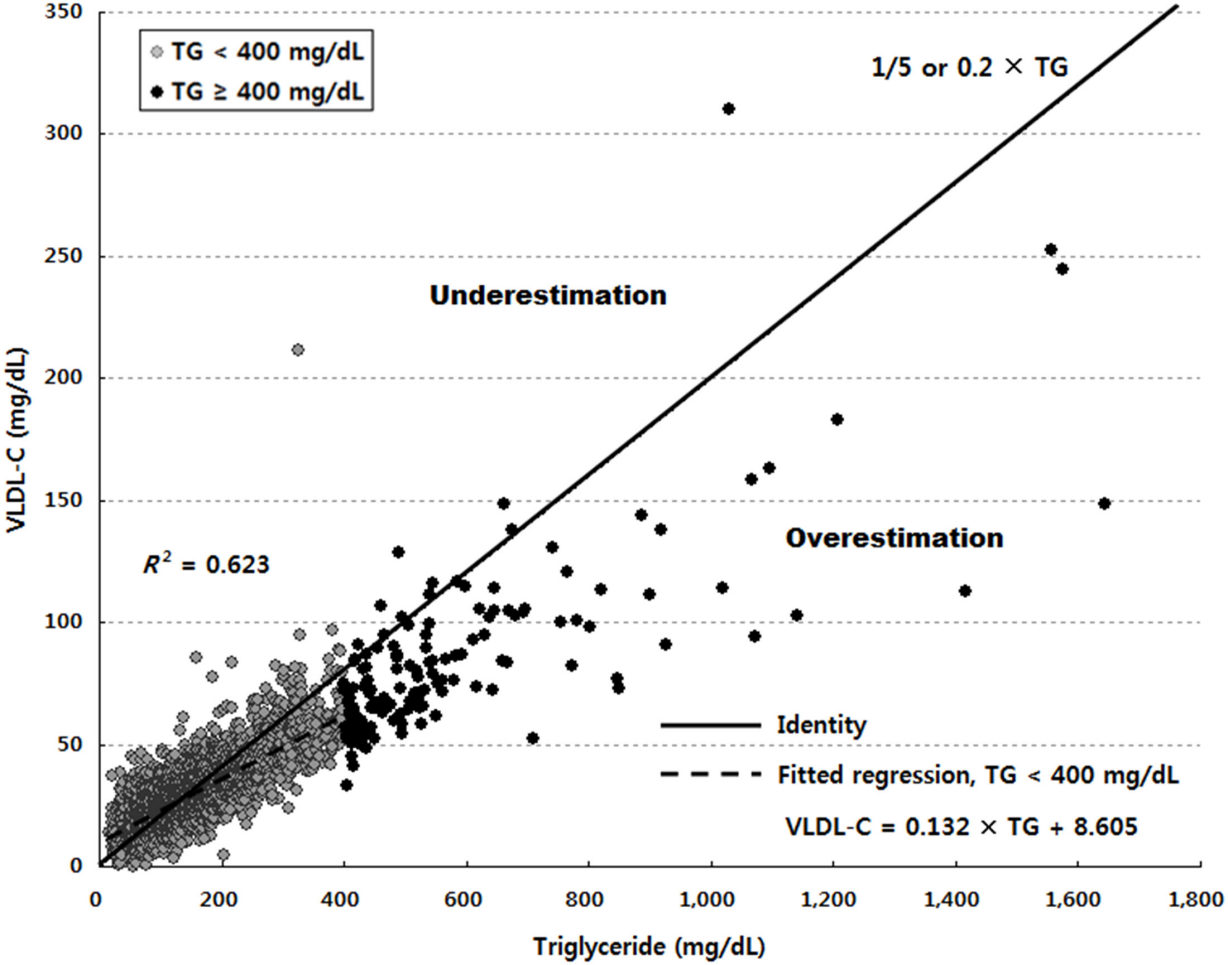
Relationship between triglyceride and very low-density lipoprotein cholesterol levels(VLDL-C indicates very low-density lipoprotein cholesterol; TG, triglyceride). The dark right-upward line represents the values of triglycerides divided by 5, the estimates of VLDL-C used in the Friedewald formula. If the true VLDL-C value is greater than the triglycerides/5 value (dots above the line), then the Friedewald equation will tend to underestimate VLDL-C, and vice versa if the true VLDL-C is less than the triglycerides/5 (dots below the line). Overall, the Friedewald formula showed a tendency to overestimate VLDL-C and thus underestimate LDL-C as triglyceride levels increased. The broken line displays the fitted regression of triglycerides on VLDL-C when triglyceride levels are lower than 400 mg/dL.

Fig 1 illustrates the relationship between triglyceride and VLDL-C values. When triglyceride levels are lower than 400 mg/dL, triglycerides explained 62.3% of the variance in VLDL-C. Adding non-HDL-C as an independent variable, as shown in Model 1 of Table 2, the explained proportion of the variance increased slightly to 64.4%. In this model, triglycerides had a partial *R*^2^ value of (0.733)^2^ = 0.537, whereas that of non-HDL-C was (0.263)^2^ = 0.069, implying that triglycerides accounted for 7.8 times more of the variance than did non-HDL-C. Controlling for non-HDL-C, HDL-C, and age in Model 2 of Table 2, triglycerides and VLDL-C showed a relatively strong relationship (partial *R* = 0.748) compared to the other independent variables.

**Table 2:** Multiple regression results using very low-density lipoprotein cholesterol (VLDL-C) as the dependent variable in 5642 Korean adults aged 20 to 87 years with triglyceride concentrations < 400 mg/dL.

Model	Dependent variable: VLDL-C					
Independent variable	*b*	(95% CI)	*p-*value	*R*	Partial *R*	Adjusted *R*^2^
**Model 1**						0.644
Triglycerides, mg/dL	0.120	(0.117, 0.123)	< 0.001	0.789	0.733	
Non-HDL-C, mg/dL	0.055	(0.049, 0.061)	< 0.001	0.480	0.263	
Constant	2.467	(1.710, 3.225)	< 0.001			
**Model 2**						0.680
Triglycerides, mg/dL	0.129	(0.126, 0.132)	< 0.001	0.789	0.748	
Non-HDL-C, mg/dL	0.045	(0.039, 0.050)	< 0.001	0.480	0.200	
HDL-C, mg/dL	0.188	(0.170, 0.206)	< 0.001	-0.181	0.267	
Age, years	0.120	(0.090, 0.115)	< 0.001	0.312	0.208	
Constant	-11.012	(-12.305, -9.718)	< 0.001			

HDL-C indicates high-density lipoprotein cholesterol; *b*, unstandardized coefficient; CI, confidence level; *R*, zero-order correlation; Partial *R*, partial correlation.

For stratification, we first used only triglycerides and then added non-HDL-C to capture each performance of the two parameters in improving LDL-C estimation. Based on strata of triglycerides, the 5-cell table is shown in Table 3. The median TG:VLDL-C ratio was significantly different among triglyceride groups (*p*< 0.001) and increased as triglyceride levels went up. The median TG:VLDL-C ratio of each triglyceride strata ranged from 2.71 to 6.21.The 25-cell table, based on strata of triglyceride and non-HDL-C values, is shown in Table 4. For the same triglyceride group, the median TG:VLDL-C ratio generally decreased as non-HDL-C levels increased.

**Table 3:** Median for the ratio of triglycerides to very low-density lipoprotein cholesterol by triglyceride strata (5-cell).

		TG:VLDL-C ratio		VLDL-C:TG ratio		
TG levels, mg/dL	*n*	Median (95% CI, Range)	*p*-value*	Mean (SD)	*p*-value^†^	T^‡^
< 50	499	2.71 (2.57–2.81, 95.1%)	< 0.001	0.398 (0.178)	< 0.001	A
50 to 99	2,105	4.11 (4.03–4.17, 95.0%)		0.255 (0.091)		B
100 to 149	1,484	5.19 (5.10–5.28, 95.4%)		0.197 (0.055)		C
150 to 199	784	5.70 (5.58–5.86, 95.6%)		0.180 (0.048)		D
200 to 399	806	6.21 (6.08–6.35, 95.5%)		0.167 (0.041)		E

TG indicates triglyceride; VLDL-C, very low-density lipoprotein cholesterol; TG:VLDL-C ratio, ratio of TG to VLDL-C; VLDL-C:TG ratio, ratio of VLDL-C to TG; CI, confidence interval; SD, standard deviation.

* *P*-value for median comparison among TG groups was calculated by median test.

^†^ *P*-value for mean comparison among TG groups was calculated by one-way analysis of variances.

^‡^ The same letters indicate non-significant difference between groups (α = 0.05) on Tamhane’s multiple comparison test.

**Table 4:** Median for the ratio of triglycerides to very low-density lipoprotein cholesterol by non-high-density lipoprotein cholesterol and triglyceride strata (25-cell).

	Non-HDL-C, mg/dL				
TG levels, mg/dL	< 100	100 to 129	130 to 159	160 to 189	≥ 190
< 50	2.8	2.7	2.5	2.7	2.1
50 to 99	4.2	4.2	4.0	3.9	3.8
100 to 149	5.5	5.5	5.1	4.9	4.5
150 to 199	6.3	6.1	5.9	5.5	4.9
200 to 399	7.1	6.7	6.4	6.2	5.5

TG indicates triglyceride; HDL-C, high-density lipoprotein cholesterol.

To generate LDL-C_N_ estimates, the strata-specific median TG: VLDL-C ratios were applied in subjects of our data set with triglyceride levels under 400 mg/dL. That is, the 5-cell table (Table 3) was used for LDL-C_5_, the 25-cell table (Table 4) for LDL-C_25_, and the 180-cell table described by Martin et al. [9] for LDL-C_180_. Comparing each estimated LDL-C and LDL-C_D_ value by the 5 strata of triglyceride levels using the Wilcoxon signed rank test (see S1 through S4 Tables), LDL-C_F_ significantly overestimated LDL-C_D_ at triglyceride levels under 100 mg/dL (*p*< 0.001) while LDL-C_F_ underestimated LDL-C_D_ at triglyceride levels of 100–399 mg/dL (*p*< 0.001). LDL-C_5_ and LDL-C_25_ did not significantly underestimate or overestimate LDL-C_D_ at any triglyceride level; however, LDL-C_180_ significantly overestimated LDL-C_D_ at all triglyceride levels (*p*< 0.001). At triglyceride levels under 400 mg/dL, LDL-C_N_ estimates were closer overall to LDL-C_D_ than LDL-C_F_ estimates (*p <* 0.001 for each comparison, see S5 Table). The median for (LDL-C_F_)–(LDL-C_D_) was 0.6 mg/dL (IQR, -5.1 to 5.7; 5th-95th percentile, -15.2 to 13.5). Examining LDL-C_N_−LDL-C_D_, the median was 0.0 mg/dL (IQR, -4.2 to 4.5; 5th-95th percentile, -9.9 to 13.2) for LDL-C_5_, 0.0 mg/dL (IQR, -4.1 to 4.4; 5th-95th percentile, -10.0 to 12.3) for LDL-C_25_, and 1.5 mg/dL (IQR, -2.5 to 5.9; 5th-95th percentile, -8.1 to 14.0) for LDL-C_180_.

Table 5 shows concordances in the NCEP-ATP III guideline classification between LDL-C estimates and LDL-C_D_ and between LDL-C estimates when triglyceride levels are lower than 400 mg/dL. Compared with LDL-C_F_, LDL-C_N_ estimates exhibited a significantly higher overall concordance (*p <* 0.001 for each comparison). In subjects with triglyceride levels of 200 to 399 mg/dL, LDL-C_N_ estimates showed significantly higher concordances than those of LDL-C_F_ estimates (*p*< 0.001 for each comparison). In particular, LDL-C_25_ provided a significantly higher concordance than LDL-C_180_ (*p* = 0.026). In other triglyceride categories, LDL-C_N_ estimates incrementally improved concordance compared with LDL-C_F_.

**Table 5:** Concordance in the NCEP-ATP III guideline classification by Friedewald vs. novel estimates of low-density lipoprotein cholesterol (LDL-C) in relation to direct LDL-C when triglycerides are lower than 400 mg/dL.

	LDL-C_F_		LDL-C_5_		LDL-C_25_		LDL-C_180_	
	C/T*	% (95% CI)	C/T	% (95% CI)	C/T	% (95% CI)	C/T	% (95% CI)
**LDL-C, mg/dL**								
≥ 190 (*n* = 89)	73/96	76.0 (67.5–84.5)	73/110	66.4 (57.6–75.2)	65/81	80.2 (71.5–88.9)	73/92	79.3 (71.0–87.6)
160 to 189 (*n* = 366)	270/350	77.1 (72.7–81.5)	272/369	73.7 (69.2–78.2)	281/357	78.7 (74.5–82.9)	291/382	76.2 (71.9–80.5)
130 to 159 (*n* = 1163)	892/1142	78.1 (75.7–80.5)	944/1166	81.0 (78.7–83.3)	962/1183	81.3 (79.1–83.5)	977/1234	79.2 (76.9–81.5)
100 to 129 (*n* = 1996)	1605/2045	78.5 (76.7–80.3)	1628/1944	83.7 (82.1–85.3)	1667/2021	82.5 (80.8–84.2)	1668/2043	82.3 (80.6–84.0)
70 to 99 (*n* = 1667)	1323/1647	80.3 (78.4–82.2)	1396/1668	83.7 (81.9–85.5)	1387/1646	84.3 (82.5–86.1)	1373/1615	85.0 (83.3–86.7)
< 70 (*n* = 361)	251/362	69.3 (64.5–74.1)	291/385	75.6 (71.3–79.9)	279/354	78.8 (74.5–83.1)	232/276	84.1 (79.8–88.4)
**Triglycerides, mg/dL**								
200 to 399	500/806	62.0 (58.6–65.4)	617/806	76.6 (73.7–79.5)	622/806	77.2 (74.3–80.1)	601/806	74.6 (71.5–77.6)
150 to 199	580/748	77.5 (74.5–80.5)	593/748	79.3 (76.4–82.2)	604/748	80.7 (77.9–83.5)	603/748	80.6 (77.8–83.4)
100 to 149	1231/1484	83.0 (81.1–84.9)	1238/1484	83.4 (81.5–85.4)	1250/1484	84.2 (82.3–86.1)	1257/1484	84.7 (82.9–86.5)
< 100	2103/2604	80.8 (79.3–82.3)	2156/2604	82.8 (81.4–84.2)	2165/2604	83.1 (81.7–84.5)	2167/2604	83.2 (81.8–84.6)
**Overall** ^†^	4414/5642	78.2 (77.1–79.3)	4604/5642	81.6 (80.6–82.6)	4641/5642	82.3 (81.3–83.3)	4628/5642	82.0 (81.0–83.0)

LDL-C indicates low-density lipoprotein cholesterol; LDL-C_F_, Friedewald LDL-C; LDL-C_5_, 5-cell method LDL-C; LDL-C_25_, 25-cell method LDL-C; LDL-C_180_, 180-cell method LDL-C (Martin et al. [9]); C/T, concordant number / total number; CI, confidence interval.

* Initial classification was defined by the LDL-C estimates: concordance was determined according to direct LDL-C.

^†^*P-*value for difference in overall concordance rates between LDL-C_F_ and each LDL-C_N_ estimate is *p*< 0.001.

**Fig 2:**
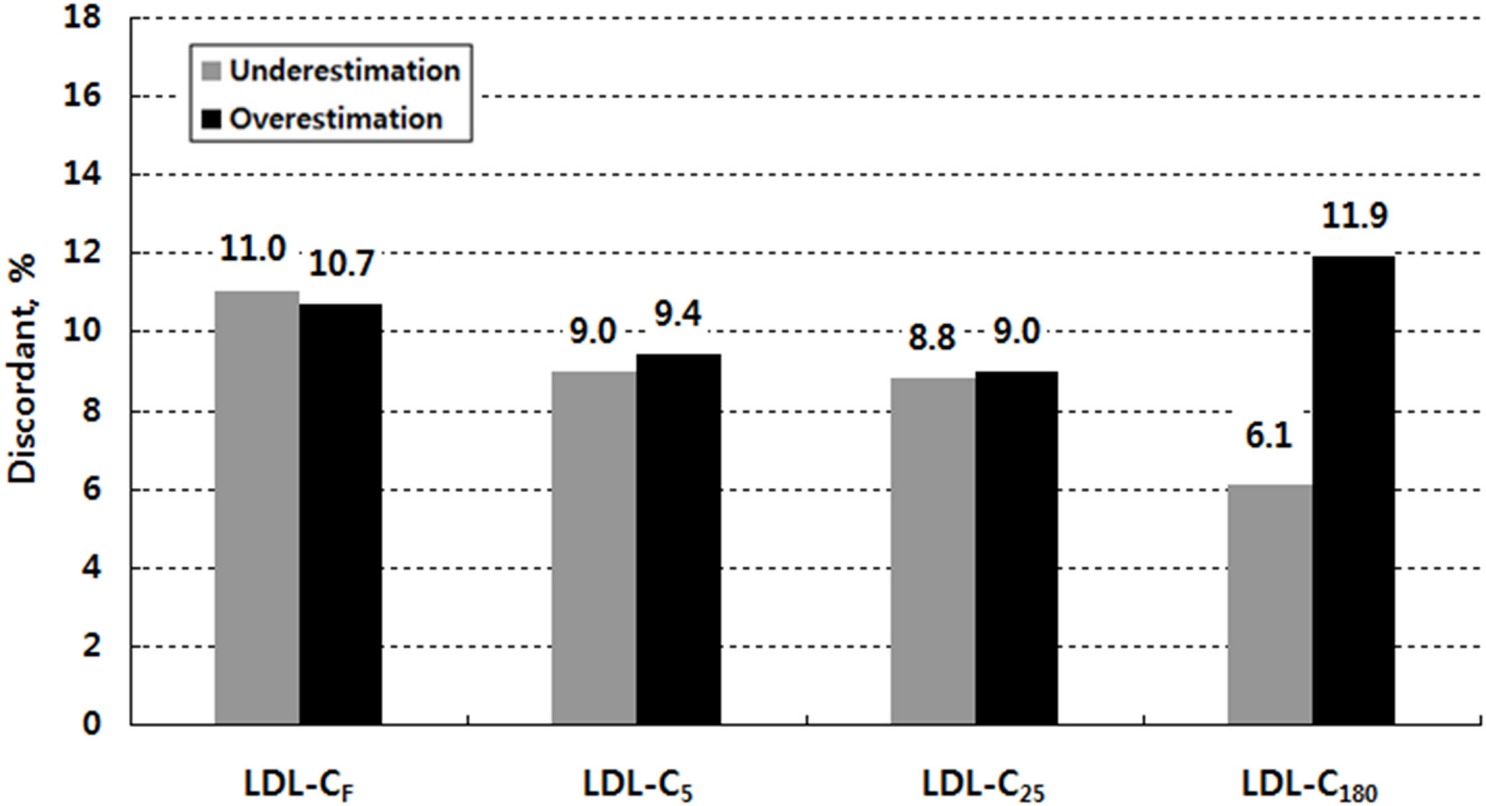
Overall discordance (underestimation vs. overestimation) in the NCEP-ATP III guideline classification by low-density lipoprotein cholesterol (LDL-C) estimate when triglycerides are lower than 400 mg/dL. LDL-C indicates low-density lipoprotein cholesterol; LDL-C_F_, Friedewald LDL-C; LDL-C_5_, 5-cell method LDL-C; LDL-C_25_, 25-cell method LDL-C; LDL-C_180_, 180-cell method LDL-C (Martin et al. [9])

Fig 2 shows overall discordance in the NCEP-ATP III guideline classification by LDL-C estimate when triglyceride levels are lower than 400 mg/dL. Within the misclassified subjects, there were two groups: those who were classified in a lower category and those who were classified in a higher category compared with their LDL-C_D_ category (see S6 Table). When we referred to the former as underestimated and the latter as overestimated in their LDL-C categories, the number of underestimated subjects was similar to that of the overestimated subjects for LDL-C_F_, LDL-C_5_, and LDL-C_25_: 623 underestimated subjects (11.0%) versus 605 overestimated subjects (10.7%) for LDL-C_F_; 509 subjects (9.0%) versus 529 subjects (9.4%) for LDL-C_5_; and 495 subjects (8.8%) versus 506 subjects (9.0%) for LDL-C_25_. For LDL-C_180_, however, the number of overestimated subjects was about two times larger than that of the underestimated subjects; 672 subjects (11.9%) were classified in a higher category, but 342 subjects (6.1%) were classified in a lower category.

## Discussion

We compared the performance of the novel method and the Friedewald formula on estimating LDL-C using a sample of 5,642 Korean adults with LDL-C measured by the enzymatic homogenous assay. As novel estimates, LDL-C_5_ and LDL-C_25_ were calculated using the strata-specific median TG:VLDL-C ratios derived from our data set, together with LDL-C_180_ using the 180-cell table described by Martin et al. [9]. Each of these LDL-C_N_ estimates was significantly more similar to LDL-C_D_ than LDL-C_F_ in subjects with triglyceride levels lower than 400 mg/dL (*p*< 0.001 for each comparison).

To estimate LDL-C, the novel method uses an adjustable factor for the TG:VLDL-C ratio according to triglyceride and non-HDL-C levels, whereas the Friedewald equation applies a fixed factor of 5. Some previous studies [12–14] have attempted to determine an optimal fixed factor. For example, DeLong et al. [12] and Puavilai et al. [13] suggested a higher fixed factor of 6 instead of 5 while Hata and Nakajima [14] proposed a lower fixed factor of 4. However, the overall median TG:VLDL-C ratio of 4.9 in our sample is closer to the original Friedewald factor of 5. In contrast, McNamara et al. [15] found that the optimal factor varied by triglyceride level and suggested different factors based on triglyceride values: 4 for triglyceride levels ≤50 mg/dL, 4.5 for levels of 51–200 mg/dL, and 5 for levels of 201–400 mg/dL. Compared with the McNamara method, the novel method extends its basis for applying the strata-specific TG:VLDL-C ratios from triglycerides to non-HDL-C values. Considering the broken line in Fig 1, the novel method is assumed to be useful for the more correct estimation of LDL-C in Korean people.

In this study, we first generated LDL-C_5_ based on only triglyceride strata. The median TG:VLDL-C ratio was significantly different among triglyceride strata (*p*< 0.001) and increased as triglyceride levels went up (Table 3), which is similar to the findings of McNamara et al. [15]. Next, we generated LDL-C_25_ based on the 25 strata of triglyceride and non-HDL-C levels. For the same triglyceride group, the median TG:VLDL-C ratio roughly decreased as non-HDL-C levels increased (Table 4), as in the 180-cell table of Martin et al. [9]. However, we were able to observe a difference between the median TG: VLDL-C ratio of LDL-C_25_ and that of LDL-C_180_ at triglyceride levels less than 50mg/dL, with ranges from 2.1 to 2.8 versus ranges from 3.1 to 3.5 for LDL-C_25_ and LDL-C_180_, respectively. Wang et al. [16] suggested that, similar to high TG levels, low TG levels affect LDL-C_F_, resulting in overestimation. They attributed this to a low-fat, low-protein diet. Considering the carbohydrate-oriented dietary life pattern of Koreans, their lipid profile may differ from that of Westerners.

The majority of previous studies have suggested that the Friedewald formula underestimates directly measured LDL-C [5–8]. In our sample, LDL-C_F_ significantly underestimated LDL-C_D_ at levels of 100–399 mg/dL, but significantly overestimated LDL-C_D_ at triglyceride levels under 100 mg/dL. On the other hand, LDL-C_180_ significantly overestimated LDL-C_D_ for the entire triglyceride range.LDL-C_5_ and LDL-C_25_ did not show any tendency of over/underestimation.

Compared with LDL-C_F_, each of the LDL-C_N_ estimates exhibited a significantly higher overall concordance with LDL-C_D_ in the NCEP-ATP III guideline classification (*p*< 0.001 for each comparison). Overall concordance was 78.2% for LDL-C_F_, 81.6% for LDL-C_5_, 82.3% for LDL-C_25_, and 82.0% for LDL-C_180_ (Table 5). Compared with LDL-C_5_, LDL-C_25_ significantly improved overall concordance (*p* = 0.008). LDL-C_25_ and LDL-C_180_ provided almost the same overall concordance. In comparison to the overall concordance of 85.4% for LDL-C_F_ versus 91.7% for LDL-C_180_ (*p*< 0.001) reported by Martin et al. [9], LDL-C_F_ and LDL-C_180_ in our sample have shown relatively low overall concordance with LDL-C_D_. Meeusen et al. [17] found that overall concordance was similar to that of our results as 76.9% [95% CI, 75.2–79.4] for LDL-C_F_ versus 77.7% [95% CI, 76.0–79.6] for LDL-C_180._In our sample, however, LDL-C_180_ has shown significantly higher overall concordance than LDL-C_F._ Compared with LDL-C_F_ by LDL-C category, LDL-C_5_ was more concordant only when LDL-C was under 160 mg/L; however, LDL-C_25_and LDL-C_180_were more concordant in all LDL-C categories. When LDL-C was lower than 70 mg/dL, as reported by Martin et al. [9], LDL-C_180_ improved greatly in concordance compared with LDL-C_F._This was particularly apparent in subjects with high triglyceride levels (see S1 Fig). In subjects with triglyceride levels of 200 to 399 mg/dL, LDL-C_25_ provided a significantly higher concordance than LDL-C_F_ (*p* = 0.001) and LDL-C_180_ (*p* = 0.026).

However, a point of precaution is that while there may be high concordance in a special stratum, the specific method in that stratum may not be the most appropriate method. LDL-C_180_ showed high concordance of 84.1% for LDL-C less than 70mg/dL, but in reality, 129 out of 361 subjects with LDL-C less than 70mg/dL showed overestimation (see Table 5, S6 Table). The overall concordance of 82.3% for LDL-C_25_ was the highest. Compared to the Friedewald formula, it showed higher concordance at all LDL-C levels, and the 78.8% concordance at LDL-C less than 70mg/dL was lower than that of LDL-C_180_, but still accurately categorized more subjects (279 individuals, Table 5). The purpose of the clinical screening test is to accurately diagnose a greater number of patients and apply appropriate treatments. Thus, simply analyzing the concordance can lead to erroneous results. Instead, multiple points must be considered, such as each method’s trend of diagnosis, sensitivity, specificity, and perceived ease of use in actual application, in addition to the concordance.

Overall, LDL-C_180_ showed approximately two times as much overestimation as underestimation (Fig 2). This result can be interpreted in two folds. First, this overestimation might be exaggerated because of problems with the method for the direct LDL measurement. Serum LDL-C concentrations in our study were directly measured using the enzymatic homogeneous assay by Sekisui Medical instead of the gold standard β-quantification reference method used by Martin et al. [9]. β-quantification consistently captures all three elements of LDL by its standard definition: true LDL plus IDL plus Lp(a). In contrast, chemical homogeneous assays directly measuring LDL-C does not consistently capture the three elements, subsequently being prone to LDL-C underestimation [5]. The overestimation observed in our study, therefore, may be actually due to the Sekisui assay missing some elements of LDL-C.

Another possible explanation is that the overestimation of LDL-C by LDL-C_180_ can be attributed to racial differences and related difference in dietary patterns. This could be postulated to impact TG:VLDL-C ratio. We have partly identified the differences in the median TG:VLDL-C ratio by triglyceride and non-HDL-C strata between Korean and American. For example, the median TG:VLDL-C ratio of subjects with TG of < 50 mg/dL and non-HDL-C of <100 mg/dL was 2.7 in our study versus 3.5 in Martin et al.’s study [9]. However, we calculated VLDL-C as non-HDL-C minus LDL-C_D,_ so it could be also affected by direct measuring method of LDL-C.

The application of strata-specific TG:VLDL-C ratios, like Martin’s novel method, can estimate a more accurate LDL-C of LDL-C levels under 70mg/dL than the Friedewald formula. In 2012, there were approximately 15 million target subjects for the Korea National Health Screening Program (KNHSP), 11.5 million of those that were actually screened, and approximately 865,000 of total screened subjects in the LDL-C<70mg/dL category [18]. In addition to subjects with TG>400mg/dL, direct measurement is needed for precise detection of the subjects with LDL-C<70mg/dL, rather than the use of Friedewald formula. However, the use of LDL-C_25_ or LDL-C_180_ allows for even more accurate estimation of LDL-C as well as the lowering of testing costs.

One of the limitations of this study is that the direct measurement method of LDL-C is not the same as β–quantification used by Martin et al. [9]. It has been reported that liquid selective detergent method, such as Sekisui Medical, revealed a negative deviation from the Friedewald’s equation and β-quantification in hypercholesterolemia [19–21]. Considering this effect for higher levels of LDL-C categories, the overestimation of LDL-C_180_ may be somewhat exaggerated than the actual value, although our study population mainly consists of normal healthy subjects. Miller et al. have compared and analyzed various homogenous assays with β-quantification [22]. In their study, Sekisui assay fulfilled the NCEP total error goal in non-diseased subjects. Other studies also have reported that some homogenous assays, including Sekisui Medical, showed a good correlation with Friedewald equation or β-quantification for common disease and healthy subjects with satisfactory precision. The authors suggested that homogenous assays could be used in epidemiologic studies, both in fasting and non-fasting samples [23,24].

Another limitation of this study is that VLDL was not directly measured, but rather it was calculated using the formula TC—HDL-C—LDL-C. In this calculation, concentrations of chylomicron, IDL-C, and others were not considered. Thus, cases such as a high level of chylomicron could increasingly lead to erroneous readings. However, the relative proportion of chylomicron in total cholesterol is very small, and all subjects were under strict eight hour fasting conditions, so the likelihood of error in this area was likely minimized as much as possible.

## Conclusions

In conclusion, Martin’s novel method, which uses the strata-specific TG:VLDL-C ratio, provides a more accurate LDL-C estimation in Koreans in comparison to the Friedewald formula, which uses the fixed TG:VLDL-C ratio. Moreover, the former showed a higher concordance in categorization based on NCEP-ATP classification. However, LDL-C_180_ based on Martin’s data, when applied to Koreans, resulted in excessive overestimation and thus may indicate racial differences in readings, but the result should be judged with considering the measuring method. It requires further investigations using the same measuring method for LDL-C. LDL-C_25_ showed the highest overall concordance of 82.3%. Most of all, in comparison to the Friedewald method, LDL-C_25_ showed higher concordance in all levels and no bias in diagnostic tendencies. Therefore, it is worth considering Martin’s novel method as a trustworthy and economical method for the estimation of LDL-C among Koreans. However, the level of racial specificity of this method as well as the specific character of the applied measuring method should be considered, and the method requires further modification and validation using strata-specific TG:VLDL-C ratios derived from data specific to Koreans.

## Supporting Information

S1 Fig Concordance of direct measurement with Friedewald and novel estimates in classifying LDL-C lower than 70 mg/dL by triglyceride strata. LDL-C indicates low-density lipoprotein cholesterol; LDL-C_F_, Friedewald LDL-C; LDL-C_5_, 5-cell method LDL-C; LDL-C_25_, 25-cell method LDL-C; LDL-C_180_, 180-cell method LDL-C (Martin et al. [9]).
(TIF)

S1 Table Results ofthe Wilcoxon signed ranks test for the median score difference between LDL-C_F_ and LDL-C_D_ values (LDL-C_F_—LDL-C_D_) by TG levels. LDL-C indicates low-density lipoprotein cholesterol; LDL-C_F_, Friedewald LDL-C; LDL-C_D_, LDL-C measured by the enzymatic homogeneous assay; TG, triglycerides. Under the null hypothesis of no difference, the sum of the ranks relating to the positive and negative difference should be the same. If SP > SN, where SP = the sum of the positive ranks and SN = the sum of the negative ranks, then LDL-C_F_ overestimates LDL-C_D_; if SN > SP, then LDL-C_F_ underestimates LDL-C_D_.
(DOCX)

S2 Table Results of the Wilcoxon signed ranks test for the median score difference between LDL-C_5_ and LDL-C_D_ values (LDL-C_5_—LDL-C_D_) by TG levels. LDL-C indicates low-density lipoprotein cholesterol; LDL-C_5_, 5-cell method LDL-C; LDL-C_D_, LDL-C measured by the enzymatic homogeneous assay; TG, triglycerides. Under the null hypothesis of no difference, the sum of the ranks relating to the positive and negative difference should be the same. If SP > SN, where SP = the sum of the positive ranks and SN = the sum of the negative ranks, then LDL-C_5_ overestimates LDL-C_D_; if SN > SP, then LDL-C_5_ underestimates LDL-C_D_.
(DOCX)

S3 Table Results ofthe Wilcoxon signed ranks test for the median score difference between LDL-C_25_ and LDL-C_D_ values (LDL-C_25_—LDL-C_D_) by TG levels. LDL-C indicates low-density lipoprotein cholesterol; LDL-C_25_, 25-cell method LDL-C; LDL-C_D_, LDL-C measured by the enzymatic homogeneous assay; TG, triglycerides. Under the null hypothesis of no difference, the sum of the ranks relating to the positive and negative difference should be the same. If SP > SN, where SP = the sum of the positive ranks and SN = the sum of the negative ranks, then LDL-C_25_ overestimates LDL-C_D_; if SN > SP, then LDL-C_25_ underestimates LDL-C_D_.
(DOCX)

S4 Table Results ofthe Wilcoxon signed ranks test for the median score difference between LDL-C_180_ and LDL-C_D_ values (LDL-C_180_—LDL-C_D_) by TG levels. LDL-C indicates low-density lipoprotein cholesterol; LDL-C_180_, 180-cell method LDL-C (Martin et al. [9]); LDL-C_D_, LDL-C measured by the enzymatic homogeneous assay; TG, triglycerides. Under the null hypothesis of no difference, the sum of the ranks relating to the positive and negative difference should be the same. If SP > SN, where SP = the sum of the positive ranks and SN = the sum of the negative ranks, then LDL-C_180_ overestimates LDL-C_D_; if SN > SP, then LDL-C_180_ underestimates LDL-C_D_.
(DOCX)

S5 Table Results of the Wilcoxon signed ranks test for the median score differences between | LDL-C_F_-LDL-C_D_| and | LDL-C_N_-LDL-C_D_|values. LDL-C indicates low-density lipoprotein cholesterol; LDL-C_F_, Friedewald LDL-C; LDL-C_5_, 5-cell method LDL-C; LDL-C_25_, 25-cell method LDL-C; LDL-C_180_, 180-cell method LDL-C (Martin et al. [9]); LDL-C_D_, LDL-C measured by the enzymatic homogeneous assay. Under the null hypothesis of no difference, the sum of the ranks relating to the positive and negative difference should be the same. If SP > SN, where SP = the sum of the positive ranks and SN = the sum of the negative ranks, then LDL-C_N_ more closely approximated LDL-C_D_; if SN > SP, then LDL-C_F_ more closely approximated LDL-C_D_.
(DOCX)

S6 Table Concordance of the NCEP-ATP III guideline classification between LDL-C_D_ and LDL-C estimates when triglycerides are lower than 400 mg/dL. LDL-C indicates low-density lipoprotein cholesterol; LDL-C_D_, LDL-C measured by the enzymatic homogeneous assay; LDL-C_F_, Friedewald LDL-C; LDL-C_5_, 5-cell method LDL-C; LDL-C_25_, 25-cell method LDL-C; LDL-C_180_, 180-cell method LDL-C (Martin et al. [9]).
(DOCX)

S7 Table Results of McNemar’s exact test for the comparison of overall concordance rates between LDL-C_F_ and each LDL-C_N_ estimate when triglycerides are lower than 400 mg/dL. LDL-C indicates low-density lipoprotein cholesterol; LDL-C_F_, Friedewald LDL-C; LDL-C_5_, 5-cell method LDL-C; LDL-C_25_, 25-cell method LDL-C; LDL-C_180_, 180-cell method LDL-C (Martin et al. [9]).
(DOCX)

S8 Table Results of McNemar’s exact test for the comparison of concordance rates between LDL-C_F_ and each LDL-C_N_ estimate by triglyceride levels. LDL-C indicates low-density lipoprotein cholesterol; LDL-C_F_, Friedewald LDL-C; LDL-C_5_, 5-cell method LDL-C; LDL-C_25_, 25-cell method LDL-C; LDL-C_180_, 180-cell method LDL-C (Martin et al. [9]); TG, triglycerides.
(DOCX)
