# Supplementary figures and images for: Validation of the Martin Method for Estimating Low-Density Lipoprotein Cholesterol Levels in Korean Adults: Findings from the Korea National Health and Nutrition Examination Survey, 2009-2011
Source: PLoS One. 2016 Jan 29;11(1):e0148147. doi: 10.1371/journal.pone.0148147 (PMC4732787; doi:10.1371/journal.pone.0148147)

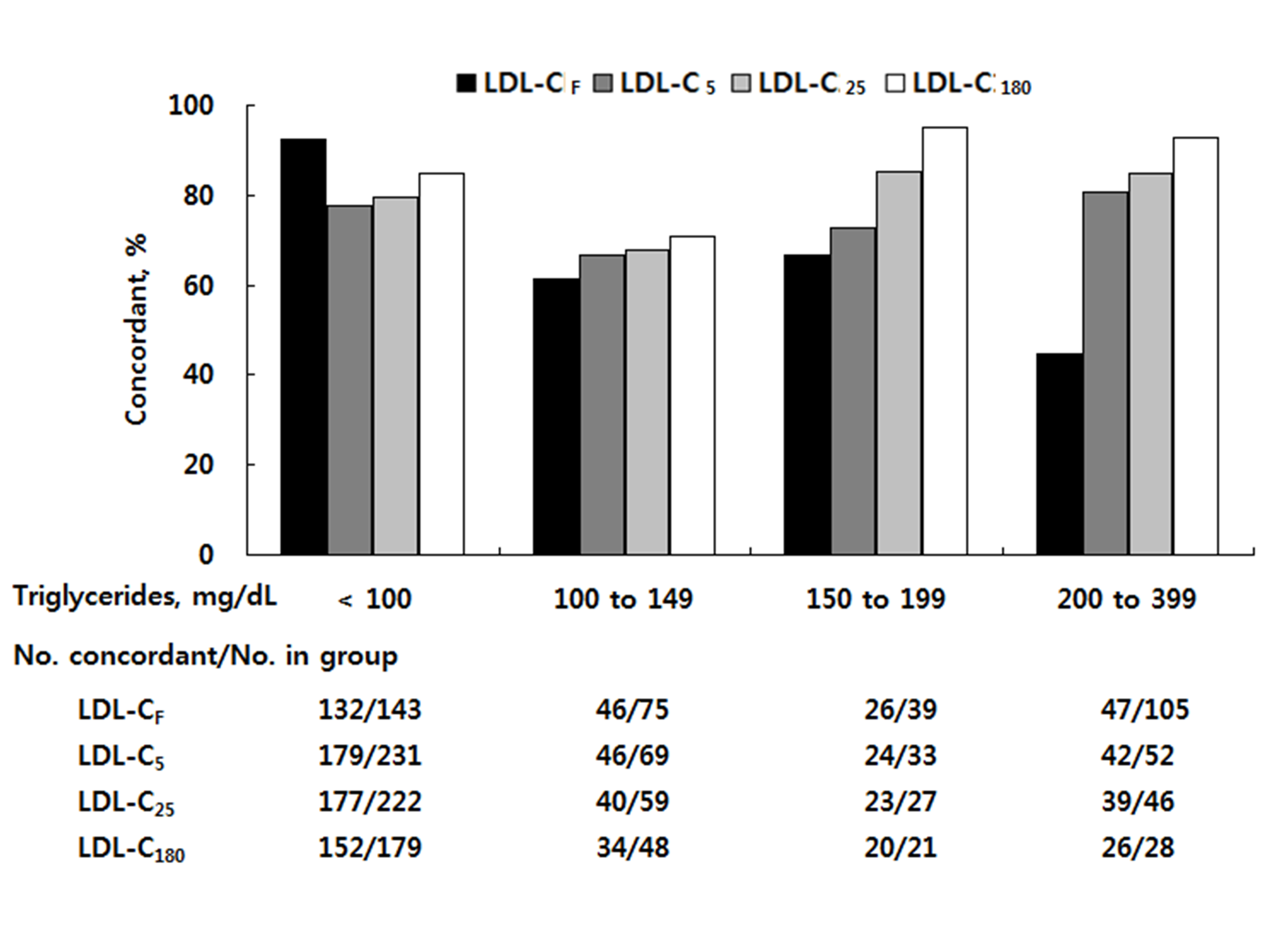

Supplement: S1 Fig — LDL-C indicates low-density lipoprotein cholesterol; LDL-CF, Friedewald LDL-C; LDL-C5, 5-cell method LDL-C; LDL-C25, 25-cell method LDL-C; LDL-C180, 180-cell method LDL-C (Martin et al. [9]). (TIF) [file pone.0148147.s001.tif]
