# Supplementary material for: Validation of the Martin Method for Estimating Low-Density Lipoprotein Cholesterol Levels in Korean Adults: Findings from the Korea National Health and Nutrition Examination Survey, 2009-2011
Source: PLoS One. 2016 Jan 29;11(1):e0148147. doi: 10.1371/journal.pone.0148147 (PMC4732787; doi:10.1371/journal.pone.0148147)
Supplement: S1 Table — LDL-C indicates low-density lipoprotein cholesterol; LDL-CF, Friedewald LDL-C; LDL-CD, LDL-C measured by the enzymatic homogeneous assay; TG, triglycerides. Under the null hypothesis of no difference, the sum of the ranks relating to the positive and negative difference should be the same. If SP > SN, where SP = the sum of the positive ranks and SN = the sum of the negative ranks, then LDL-CF overestimates LDL-CD; if SN > SP, then LDL-CF underestimates LDL-CD. (DOCX) [file pone.0148147.s002.docx]

**S1 Table.** Results of the Wilcoxon signed ranks test for the median score difference between LDL-C_F_ and LDL-C_D_ values (LDL-C_F_ - LDL-C_D_) by TG levels

| **TG levels, mg/dL** | **Signed ranks** | ***n*** | **Mean rank** | **Sum of ranks** | **Z** | ***p*-value** |
| --- | --- | --- | --- | --- | --- | --- |
| < 50 | Negative ranks | 48 | 77.29 | 3710.00 | -18.204 | < 0.001 |
|  | Positive ranks | 451 | 268.38 | 121040.00 |  |  |
|  | Ties | 0 |  |  |  |  |
|  | Total | 499 |  |  |  |  |
| 50 to 99 | Negative ranks | 559 | 754.15 | 421572.00 | -24.521 | < 0.001 |
|  | Positive ranks | 1541 | 1158.00 | 1784478.00 |  |  |
|  | Ties | 5 |  |  |  |  |
|  | Total | 2105 |  |  |  |  |
| 100 to 149 | Negative ranks | 823 | 755.87 | 622080.50 | -4.309 | < 0.001 |
|  | Positive ranks | 661 | 725.85 | 479789.50 |  |  |
|  | Ties | 0 |  |  |  |  |
|  | Total | 1484 |  |  |  |  |
| 150 to 199 | Negative ranks | 531 | 396.35 | 210464.50 | -11.997 | < 0.001 |
|  | Positive ranks | 216 | 319.04 | 68913.50 |  |  |
|  | Ties | 1 |  |  |  |  |
|  | Total | 748 |  |  |  |  |
| 200 to 399 | Negative ranks | 668 | 435.08 | 290632.50 | -19.363 | < 0.001 |
|  | Positive ranks | 138 | 250.64 | 34588.50 |  |  |
|  | Ties | 0 |  |  |  |  |
|  | Total | 806 |  |  |  |  |

LDL-C indicates low-density lipoprotein cholesterol; LDL-C_F_, Friedewald LDL-C; LDL-C_D_, LDL-C measured by the enzymatic homogeneous assay; TG, triglycerides.
