# Supplementary material for: Validation of the Martin Method for Estimating Low-Density Lipoprotein Cholesterol Levels in Korean Adults: Findings from the Korea National Health and Nutrition Examination Survey, 2009-2011
Source: PLoS One. 2016 Jan 29;11(1):e0148147. doi: 10.1371/journal.pone.0148147 (PMC4732787; doi:10.1371/journal.pone.0148147)
Supplement: S2 Table — LDL-C indicates low-density lipoprotein cholesterol; LDL-C5, 5-cell method LDL-C; LDL-CD, LDL-C measured by the enzymatic homogeneous assay; TG, triglycerides. Under the null hypothesis of no difference, the sum of the ranks relating to the positive and negative difference should be the same. If SP > SN, where SP = the sum of the positive ranks and SN = the sum of the negative ranks, then LDL-C5 overestimates LDL-CD; if SN > SP, then LDL-C5 underestimates LDL-CD. (DOCX) [file pone.0148147.s003.docx]

**S2 Table.** Results of the Wilcoxon signed ranks test for the median score difference between LDL-C_5_ and LDL-C_D_ values (LDL-C_5_ - LDL-C_D_) by TG levels

| **TG levels, mg/dL** | **Signed ranks** | ***n*** | **Mean rank** | **Sum of ranks** | **Z** | ***p*-value** |
| --- | --- | --- | --- | --- | --- | --- |
| < 50 | Negative ranks | 250 | 233.74 | 58434.00 | -1.223 | 0.221 |
|  | Positive ranks | 249 | 266.33 | 66316.00 |  |  |
|  | Ties | 0 |  |  |  |  |
|  | Total | 499 |  |  |  |  |
| 50 to 99 | Negative ranks | 1059 | 1010.91 | 1070555.00 | -1.353 | 0.176 |
|  | Positive ranks | 1046 | 1095.61 | 1146011.00 |  |  |
|  | Ties | 0 |  |  |  |  |
|  | Total | 2105 |  |  |  |  |
| 100 to 149 | Negative ranks | 740 | 718.86 | 531954.00 | -1.150 | 0.250 |
|  | Positive ranks | 744 | 766.02 | 569916.00 |  |  |
|  | Ties | 0 |  |  |  |  |
|  | Total | 1484 |  |  |  |  |
| 150 to 199 | Negative ranks | 374 | 360.67 | 134891.50 | -0.875 | 0.382 |
|  | Positive ranks | 374 | 388.33 | 145234.50 |  |  |
|  | Ties | 0 |  |  |  |  |
|  | Total | 748 |  |  |  |  |
| 200 to 399 | Negative ranks | 405 | 378.65 | 153352.50 | -1.400 | 0.161 |
|  | Positive ranks | 401 | 428.60 | 171868.50 |  |  |
|  | Ties | 0 |  |  |  |  |
|  | Total | 806 |  |  |  |  |

LDL-C indicates low-density lipoprotein cholesterol; LDL-C_5_, 5-cell method LDL-C; LDL-C_D_, LDL-C measured by the enzymatic homogeneous assay; TG, triglycerides.
