# Supplementary material for: Validation of the Martin Method for Estimating Low-Density Lipoprotein Cholesterol Levels in Korean Adults: Findings from the Korea National Health and Nutrition Examination Survey, 2009-2011
Source: PLoS One. 2016 Jan 29;11(1):e0148147. doi: 10.1371/journal.pone.0148147 (PMC4732787; doi:10.1371/journal.pone.0148147)
Supplement: S3 Table — LDL-C indicates low-density lipoprotein cholesterol; LDL-C25, 25-cell method LDL-C; LDL-CD, LDL-C measured by the enzymatic homogeneous assay; TG, triglycerides. Under the null hypothesis of no difference, the sum of the ranks relating to the positive and negative difference should be the same. If SP > SN, where SP = the sum of the positive ranks and SN = the sum of the negative ranks, then LDL-C25 overestimates LDL-CD; if SN > SP, then LDL-C25 underestimates LDL-CD. (DOCX) [file pone.0148147.s004.docx]

**S3 Table.** Results of the Wilcoxon signed ranks test for the median score difference between LDL-C_25_ and LDL-C_D_ values (LDL-C_25_ - LDL-C_D_) by TG levels

| **TG levels, mg/dL** | **Signed ranks** | ***n*** | **Mean rank** | **Sum of ranks** | **Z** | ***p*-value** |
| --- | --- | --- | --- | --- | --- | --- |
| < 50 | Negative ranks | 249 | 236.71 | 58941.00 | -1.066 | 0.287 |
|  | Positive ranks | 250 | 263.24 | 65809.00 |  |  |
|  | Ties | 0 |  |  |  |  |
|  | Total | 499 |  |  |  |  |
| 50 to 99 | Negative ranks | 1049 | 1017.81 | 1067682.50 | -1.456 | 0.145 |
|  | Positive ranks | 1056 | 1087.96 | 1148882.50 |  |  |
|  | Ties | 0 |  |  |  |  |
|  | Total | 2105 |  |  |  |  |
| 100 to 149 | Negative ranks | 754 | 741.58 | 559151.00 | -0.498 | 0.619 |
|  | Positive ranks | 730 | 743.45 | 542719.00 |  |  |
|  | Ties | 0 |  |  |  |  |
|  | Total | 1484 |  |  |  |  |
| 150 to 199 | Negative ranks | 369 | 363.87 | 134267.50 | -0.980 | 0.327 |
|  | Positive ranks | 379 | 384.85 | 145858.50 |  |  |
|  | Ties | 0 |  |  |  |  |
|  | Total | 748 |  |  |  |  |
| 200 to 399 | Negative ranks | 398 | 385.20 | 153309.50 | -1.407 | 0.160 |
|  | Positive ranks | 408 | 421.35 | 171911.50 |  |  |
|  | Ties | 0 |  |  |  |  |
|  | Total | 806 |  |  |  |  |

LDL-C indicates low-density lipoprotein cholesterol; LDL-C_25_, 25-cell method LDL-C; LDL-C_D_, LDL-C measured by the enzymatic homogeneous assay; TG, triglycerides.
