# Supplementary material for: Validation of the Martin Method for Estimating Low-Density Lipoprotein Cholesterol Levels in Korean Adults: Findings from the Korea National Health and Nutrition Examination Survey, 2009-2011
Source: PLoS One. 2016 Jan 29;11(1):e0148147. doi: 10.1371/journal.pone.0148147 (PMC4732787; doi:10.1371/journal.pone.0148147)
Supplement: S4 Table — LDL-C indicates low-density lipoprotein cholesterol; LDL-C180, 180-cell method LDL-C (Martin et al. [9]); LDL-CD, LDL-C measured by the enzymatic homogeneous assay; TG, triglycerides. Under the null hypothesis of no difference, the sum of the ranks relating to the positive and negative difference should be the same. If SP > SN, where SP = the sum of the positive ranks and SN = the sum of the negative ranks, then LDL-C180 overestimates LDL-CD; if SN > SP, then LDL-C180 underestimates LDL-CD. (DOCX) [file pone.0148147.s005.docx]

**S4 Table.** Results of the Wilcoxon signed ranks test for the median score difference between LDL-C_180_ and LDL-C_D_ values (LDL-C_180_ - LDL-C_D_) by TG levels

| **TG levels, mg/dL** | **Signed ranks** | ***n*** | **Mean rank** | **Sum of ranks** | **Z** | ***p*-value** |
| --- | --- | --- | --- | --- | --- | --- |
| < 50 | Negative ranks | 140 | 173.67 | 24313.50 | -11.811 | < 0.001 |
|  | Positive ranks | 359 | 279.77 | 100436.50 |  |  |
|  | Ties | 0 |  |  |  |  |
|  | Total | 499 |  |  |  |  |
| 50 to 99 | Negative ranks | 800 | 888.57 | 710855.50 | -14.250 | < 0.001 |
|  | Positive ranks | 1305 | 1153.80 | 1505709.50 |  |  |
|  | Ties | 0 |  |  |  |  |
|  | Total | 2105 |  |  |  |  |
| 100 to 149 | Negative ranks | 696 | 699.55 | 486884.50 | -3.879 | < 0.001 |
|  | Positive ranks | 788 | 780.44 | 614985.50 |  |  |
|  | Ties | 0 |  |  |  |  |
|  | Total | 1484 |  |  |  |  |
| 150 to 199 | Negative ranks | 342 | 340.10 | 116314.50 | -4.017 | < 0.001 |
|  | Positive ranks | 406 | 403.48 | 163811.50 |  |  |
|  | Ties | 0 |  |  |  |  |
|  | Total | 748 |  |  |  |  |
| 200 to 399 | Negative ranks | 270 | 331.62 | 89536.50 | -11.052 | < 0.001 |
|  | Positive ranks | 536 | 439.71 | 235684.50 |  |  |
|  | Ties | 0 |  |  |  |  |
|  | Total | 806 |  |  |  |  |

LDL-C indicates low-density lipoprotein cholesterol; LDL-C_180_, 180-cell method LDL-C (Martin et al. [9]); LDL-C_D_, LDL-C measured by the enzymatic homogeneous assay; TG, triglycerides.
