# Supplementary material for: Validation of the Martin Method for Estimating Low-Density Lipoprotein Cholesterol Levels in Korean Adults: Findings from the Korea National Health and Nutrition Examination Survey, 2009-2011
Source: PLoS One. 2016 Jan 29;11(1):e0148147. doi: 10.1371/journal.pone.0148147 (PMC4732787; doi:10.1371/journal.pone.0148147)
Supplement: S5 Table — LDL-C indicates low-density lipoprotein cholesterol; LDL-CF, Friedewald LDL-C; LDL-C5, 5-cell method LDL-C; LDL-C25, 25-cell method LDL-C; LDL-C180, 180-cell method LDL-C (Martin et al. [9]); LDL-CD, LDL-C measured by the enzymatic homogeneous assay. Under the null hypothesis of no difference, the sum of the ranks relating to the positive and negative difference should be the same. If SP > SN, where SP = the sum of the positive ranks and SN = the sum of the negative ranks, then LDL-CN more closely approximated LDL-CD; if SN > SP, then LDL-CF more closely approximated LDL-CD. (DOCX) [file pone.0148147.s006.docx]

**S5 Table.** Results of the Wilcoxon signed ranks test for the median score differences between | LDL-C_F_-LDL-C_D_| and | LDL-C_N_-LDL-C_D_| values

|  | **Signed ranks** | ***n*** | **Mean rank** | **Sum of ranks** | **Z** | ***p*-value** |
| --- | --- | --- | --- | --- | --- | --- |
| \| LDL-C_F_-LDL-C_D_\|  -\| LDL-C_5_-LDL-C_D_\| | Negative ranks | 2195 | 2543.66 | 5491770.00 | -20.168 | < 0.001 |
|  | Positive ranks | 3483 | 2993.72 | 10427113.00 |  |  |
|  | Ties | 0 |  |  |  |  |
|  | Total | 5642 |  |  |  |  |
| \| LDL-C_F_-LDL-C_D_\|  -\| LDL-C_25_-LDL-C_D_\| | Negative ranks | 2118 | 2520.22 | 5337820.50 | -21.427 | < 0.001 |
|  | Positive ranks | 3524 | 3002.58 | 10581082.50 |  |  |
|  | Ties | 0 |  |  |  |  |
|  | Total | 5642 |  |  |  |  |
| \| LDL-C_F_-LDL-C_D_\|  -\| LDL-C_180_-LDL-C_D_\| | Negative ranks | 1840 | 2438.11 | 4486114.00 | -23.002 | < 0.001 |
|  | Positive ranks | 3474 | 2773.70 | 9635841.00 |  |  |
|  | Ties | 328 |  |  |  |  |
|  | Total | 5642 |  |  |  |  |

LDL-C indicates low-density lipoprotein cholesterol; LDL-C_F_, Friedewald LDL-C; LDL-C_5_, 5-cell method LDL-C; LDL-C_25_, 25-cell method LDL-C; LDL-C_180_, 180-cell method LDL-C (Martin et al. [9]); LDL-C_D_, LDL-C measured by the enzymatic homogeneous assay.
