# Supplementary material for: Validation of the Martin Method for Estimating Low-Density Lipoprotein Cholesterol Levels in Korean Adults: Findings from the Korea National Health and Nutrition Examination Survey, 2009-2011
Source: PLoS One. 2016 Jan 29;11(1):e0148147. doi: 10.1371/journal.pone.0148147 (PMC4732787; doi:10.1371/journal.pone.0148147)
Supplement: S6 Table — LDL-C indicates low-density lipoprotein cholesterol; LDL-CD, LDL-C measured by the enzymatic homogeneous assay; LDL-CF, Friedewald LDL-C; LDL-C5, 5-cell method LDL-C; LDL-C25, 25-cell method LDL-C; LDL-C180, 180-cell method LDL-C (Martin et al. [9]). (DOCX) [file pone.0148147.s007.docx]

**S6 Table.** Concordance of the NCEP-ATP III guideline classification between LDL-C_D_ and LDL-C estimates when triglycerides are lower than 400 mg/dL

| **LDL-C estimate levels,**  **mg/dL** | **Direct LDL-C levels, mg/dL** | | | | | | |
| --- | --- | --- | --- | --- | --- | --- | --- |
|  | < 70 | 70 to 99 | 100 to 129 | 130 to 159 | 160 to 189 | ≥ 190 | Total |
| **LDL-C_F_** |  |  |  |  |  |  |  |
| < 70 | 251 | 111 | 0 | 0 | 0 | 0 | 362 |
| 70 to 99 | 108 | 1323 | 216 | 0 | 0 | 0 | 1647 |
| 100 to 129 | 2 | 232 | 1605 | 206 | 0 | 0 | 2045 |
| 130 to 159 | 0 | 1 | 175 | 892 | 74 | 0 | 1142 |
| 160 to 189 | 0 | 0 | 0 | 64 | 270 | 16 | 350 |
| ≥ 190 | 0 | 0 | 0 | 1 | 22 | 73 | 96 |
| **LDL-C_5_** |  |  |  |  |  |  |  |
| < 70 | 291 | 94 | 0 | 0 | 0 | 0 | 385 |
| 70 to 99 | 69 | 1396 | 203 | 0 | 0 | 0 | 1668 |
| 100 to 129 | 1 | 177 | 1628 | 138 | 0 | 0 | 1944 |
| 130 to 159 | 0 | 0 | 164 | 944 | 58 | 0 | 1166 |
| 160 to 189 | 0 | 0 | 1 | 80 | 272 | 16 | 369 |
| ≥ 190 | 0 | 0 | 0 | 1 | 36 | 73 | 110 |
| **LDL-C_25_** |  |  |  |  |  |  |  |
| < 70 | 279 | 75 | 0 | 0 | 0 | 0 | 354 |
| 70 to 99 | 81 | 1387 | 178 | 0 | 0 | 0 | 1646 |
| 100 to 129 | 1 | 205 | 1667 | 148 | 0 | 0 | 2021 |
| 130 to 159 | 0 | 0 | 151 | 962 | 70 | 0 | 1183 |
| 160 to 189 | 0 | 0 | 0 | 52 | 281 | 24 | 357 |
| ≥ 190 | 0 | 0 | 0 | 1 | 15 | 65 | 81 |
| **LDL-C_180_** |  |  |  |  |  |  |  |
| < 70 | 232 | 44 | 0 | 0 | 0 | 0 | 276 |
| 70 to 99 | 127 | 1373 | 115 | 0 | 0 | 0 | 1615 |
| 100 to 129 | 2 | 249 | 1682 | 110 | 0 | 0 | 2043 |
| 130 to 159 | 0 | 1 | 199 | 977 | 57 | 0 | 1234 |
| 160 to 189 | 0 | 0 | 0 | 75 | 291 | 16 | 382 |
| ≥ 190 | 0 | 0 | 0 | 1 | 18 | 73 | 92 |

LDL-C indicates low-density lipoprotein cholesterol; LDL-C_D_, LDL-C measured by the enzymatic homogeneous assay; LDL-C_F_, Friedewald LDL-C; LDL-C_5_, 5-cell method LDL-C; LDL-C_25_, 25-cell method LDL-C; LDL-C_180_, 180-cell method LDL-C (Martin et al. [9]).
