# Supplementary material for: Validation of the Martin Method for Estimating Low-Density Lipoprotein Cholesterol Levels in Korean Adults: Findings from the Korea National Health and Nutrition Examination Survey, 2009-2011
Source: PLoS One. 2016 Jan 29;11(1):e0148147. doi: 10.1371/journal.pone.0148147 (PMC4732787; doi:10.1371/journal.pone.0148147)
Supplement: S7 Table — LDL-C indicates low-density lipoprotein cholesterol; LDL-CF, Friedewald LDL-C; LDL-C5, 5-cell method LDL-C; LDL-C25, 25-cell method LDL-C; LDL-C180, 180-cell method LDL-C (Martin et al. [9]). (DOCX) [file pone.0148147.s008.docx]

**S7 Table.** Results of McNemar’s exact test for the comparison of overall concordance rates between LDL-C_F_ and each LDL-C_N_ estimate when triglycerides are lower than 400 mg/dL

| **LDL-C_N_ estimate** | **Outcome** | **Outcome of LDL-C_F_** | | *p*-value |
| --- | --- | --- | --- | --- |
|  |  | Concordance | Discordance |  |
|  |  | *n* (%) | *n* (%) |  |
| **LDL-C_5_** | Concordance | 4111 (72.9) | 493 (8.7) | < 0.001 |
|  | Discordance | 303 (5.4) | 735 (13.0) |  |
| **LDL-C_25_** | Concordance | 4127 (73.1) | 514 (9.1) | < 0.001 |
|  | Discordance | 287 (5.1) | 714 (12.7) |  |
| **LDL-C_180_** | Concordance | 4149 (73.5) | 479 (8.5) | < 0.001 |
|  | Discordance | 265 (4.7) | 749 (13.3) |  |

LDL-C indicates low-density lipoprotein cholesterol; LDL-C_F_, Friedewald LDL-C; LDL-C_5_, 5-cell method LDL-C; LDL-C_25_, 25-cell method LDL-C; LDL-C_180_, 180-cell method LDL-C (Martin et al. [9]).
