# Supplementary material for: Validation of the Martin Method for Estimating Low-Density Lipoprotein Cholesterol Levels in Korean Adults: Findings from the Korea National Health and Nutrition Examination Survey, 2009-2011
Source: PLoS One. 2016 Jan 29;11(1):e0148147. doi: 10.1371/journal.pone.0148147 (PMC4732787; doi:10.1371/journal.pone.0148147)
Supplement: S8 Table — LDL-C indicates low-density lipoprotein cholesterol; LDL-CF, Friedewald LDL-C; LDL-C5, 5-cell method LDL-C; LDL-C25, 25-cell method LDL-C; LDL-C180, 180-cell method LDL-C (Martin et al. [9]); TG, triglycerides. (DOCX) [file pone.0148147.s009.docx]

**S8 Table.** Results of McNemar’s exact test for the comparison of concordance rates between LDL-C_F_ and each LDL-C_N_ estimate by triglyceride levels

| **LDL-C_N_ estimate** | **TG levels, mg/dL** | **Outcome** | **Outcome of LDL-C_F_** | | *p*-value |
| --- | --- | --- | --- | --- | --- |
|  |  |  | Concordance | Discordance |  |
|  |  |  | *n* (%) | *n* (%) |  |
| **LDL-C_5_** | < 100 | Concordance | 1940 (74.5) | 216 (8.3) | 0.007 |
|  |  | Discordance | 163 (6.3) | 285 (10.9) |  |
|  | 100 to 149 | Concordance | 1209 (81.5) | 29 (2.0) | 0.401 |
|  |  | Discordance | 22 (1.5) | 224 (15.1) |  |
|  | 150 to 199 | Concordance | 540 (72.2) | 53 (7.1) | 0.213 |
|  |  | Discordance | 40 (5.3) | 115 (15.4) |  |
|  | 200 to 399 | Concordance | 422 (52.4) | 195 (24.2) | < 0.001 |
|  |  | Discordance | 78 (9.7) | 111 (13.8) |  |
| **LDL-C_25_** | < 100 | Concordance | 1949 (74.8) | 216 (8.3) | 0.001 |
|  |  | Discordance | 154 (5.9) | 285 (10.9) |  |
|  | 100 to 149 | Concordance | 1203 (81.1) | 47 (3.2) | 0.037 |
|  |  | Discordance | 28 (1.9) | 206 (13.9) |  |
|  | 150 to 199 | Concordance | 543 (72.6) | 61 (8.2) | 0.020 |
|  |  | Discordance | 37 (4.9) | 107 (14.3) |  |
|  | 200 to 399 | Concordance | 432 (53.6) | 190 (23.6) | < 0.001 |
|  |  | Discordance | 68 (8.4) | 116 (14.4) |  |
| **LDL-C_180_** | < 100 | Concordance | 2039 (78.3) | 128 (4.9) | < 0.001 |
|  |  | Discordance | 64 (2.5) | 373 (14.3) |  |
|  | 100 to 149 | Concordance | 1197 (80.7) | 60 (4.0) | 0.010 |
|  |  | Discordance | 34 (2.3) | 193 (13.0) |  |
|  | 150 to 199 | Concordance | 529 (70.7) | 74 (9.9) | 0.049 |
|  |  | Discordance | 51 (6.8) | 94 (12.6) |  |
|  | 200 to 399 | Concordance | 384 (47.6) | 217 (26.9) | < 0.001 |
|  |  | Discordance | 116 (14.4) | 89 (11.0) |  |

TG indicates triglyceride; LDL-C, low-density lipoprotein cholesterol; LDL-C_F_, Friedewald LDL-C; LDL-C_5_, 5-cell method LDL-C; LDL-C_25_, 25-cell method LDL-C; LDL-C_180_, 180-cell method LDL-C (Martin et al. [9]).
